# The Usefulness of Assessing Heart Rate Variability in Patients with Acute Myocardial Infarction (HeaRt-V-AMI)

**DOI:** 10.3390/s22093571

**Published:** 2022-05-07

**Authors:** Crischentian Brinza, Mariana Floria, Adrian Covic, Andreea Covic, Dragos-Viorel Scripcariu, Alexandru Burlacu

**Affiliations:** 1 Faculty of Medicine, University of Medicine and Pharmacy “Grigore T Popa”, 700115 Iasi, Romania; crischentian.brinza@d.umfiasi.ro (C.B.); floria.mariana@umfiasi.ro (M.F.); adrian.covic@umfiasi.ro (A.C.); andreea.covic@d.umfiasi.ro (A.C.); alexandru.burlacu@umfiasi.ro (A.B.); 2 Department of Interventional Cardiology, Institute of Cardiovascular Diseases “Prof. Dr. George I.M. Georgescu”, 700503 Iasi, Romania; 3 Internal Medicine Clinic, “Dr. Iacob Czihac” Military Emergency Clinical Hospital, 700483 Iasi, Romania; 4 Dialysis and Renal Transplant Center, Nephrology Clinic, “C.I. Parhon” University Hospital, 700503 Iasi, Romania

**Keywords:** ST elevation myocardial infarction, heart rate variability, percutaneous coronary intervention, prognosis, prospective study, risk factors, autonomic nervous system, wearable electronic devices

## Abstract

Background: Heart rate variability (HRV) could have independent and critical prognostic values in patients admitted for ST segment elevation myocardial infarction (STEMI). There are limited data in the literature regarding HRV assessment in STEMI setting. Thus, we aim to investigate the potential correlations between HRV and adverse outcomes in a contemporary cohort of patients presenting with STEMI undergoing primary percutaneous coronary intervention (PCI). Methods: We will perform a prospective, observational cohort study in a single healthcare center. Adult patients aged ≥18 years presenting with STEMI in sinus rhythm will be enrolled for primary PCI within 12 h from symptoms onset. Time domain, frequency domain, and nonlinear HRV parameters will be measured using a medically approved wrist-wearable device for 5 min segments during myocardial revascularization by primary PCI. Additional HRV measurements will be performed one and six months from the index event. The primary composite outcome will include all-cause mortality and major adverse cardiovascular events (during the hospital stay, one month, and one year following admission). Several secondary outcomes will be analyzed: individual components of the primary composite outcome, target lesion revascularization, hospitalizations for heart failure, ventricular arrhythmias, left ventricular ejection fraction, and left ventricular diastolic function. Conclusions: Our study will enlighten the reliability and usefulness of HRV evaluation as a prognostic marker in contemporary patients with STEMI. The potential validation of HRV as a risk marker for adverse outcomes following STEMI will ensure a background for including HRV parameters in future risk scores and guidelines.

## 1. Introduction

Ischemic heart disease represents the leading global cause of death, owing to a higher prevalence of risk factors, such as arterial hypertension, diabetes mellitus, metabolic syndrome, and aging [1]. Hence, ischemic heart disease patients with an increased risk of adverse outcomes should be identified early to ensure a close follow-up and a timely therapeutic intervention.

In the case of patients with ST segment elevation myocardial infarction (STEMI), the European Society of Cardiology (ESC) guidelines advocated for early assessment of short- and long-term risks of adverse events [2]. In this regard, the Global Registry of Acute Coronary Events (GRACE) score was endorsed to identify high-risk patients [2,3]. Heart rate variability (HRV) is an indirect marker of autonomic nervous system function. To date, HRV proves to be an excellent prognostic element for mortality in patients with acute myocardial infarction or end-stage kidney disease [4,5]. However, none of the existing risk scores is perfect in predicting subsequent worse outcomes following STEMI. Therefore, the ability of HRV to increase the prediction power of current clinical models in STEMI is a matter of further research.

In addition to clinical, biological, and imagistic parameters recommended by the guidelines for risk stratification, HRV could have a significant and independent prognostic value in STEMI patients [6]. HRV represents the fluctuation in time of RR intervals, reflecting the balance between sympathetic and parasympathetic autonomic nervous systems [7]. In addition to neural activities, respiratory rate could also influence HRV measurements [8,9]. In addition, different respiratory biofeedback techniques were developed to modulate HRV parameters [10]. HRV should not be regarded as a single parameter, as it could be assessed by different time domain, frequency domain, and nonlinear measurements [11].

An early study revealed an association between HRV and mortality in patients with acute myocardial infarction (AMI), including those treated with thrombolysis (*p* = 0.028) [4]. In a more recent trial involving STEMI patients (with reperfusion therapy), time domain HRV measurements were associated with all-cause mortality [12]. However, there were no documented differences in HRV parameters regarding other end-points, including cardiac mortality and major clinical events [12].

Thus, the relevance of HRV assessment in a contemporary cohort of patients presenting with STEMI should be investigated, especially in the era of the new generation of drug-eluting stents.

Although most studies measured HRV parameters during 24 h electrocardiographic monitoring, some parameters are validated for a shorter time interval (2–5 min) [7,11]. This is of particular interest, as wearable devices were developed to provide (short-timed) HRV data and already received CE marks for medical use [13,14,15]. As far as we know, the utility of HRV assessment using wearable devices in STEMI patients was never investigated in clinical studies.

Therefore, we will conduct a single-center, prospective, observational cohort study to investigate the prognostic role of HRV recordings in patients presenting with STEMI revascularized by primary percutaneous coronary intervention (PCI).

## 2. Materials and Methods

We designed a protocol of observational, prospective cohort study according to STROBE statement (see Table S1), in which patients with STEMI will be enrolled [16]. At least 200 consecutive patients who meet inclusion criteria referred to the catheterization laboratory from the Institute of Cardiovascular Diseases “Prof. George I.M. Georgescu” will be analyzed. The study protocol was registered in the ClinicalTrials.gov database (NCT05098977) and was approved by the Ethics Committee of the Institute of Cardiovascular Diseases “Prof. George I.M. Georgescu” in Iasi, Romania.

Interventional studies involving animals or humans, and other studies that require ethical approval, must list the authority that provided approval and the corresponding ethical approval code.

### 2.1. Study Objectives

The objectives of our study are: (1) HRV measurement in patients presenting with STEMI revascularized by primary PCI using a wearable medical device; (2) Correlation assessment between HRV and short- and long-term adverse clinical events, including different subgroups of patients (chronic kidney disease, diabetes mellitus, elderly); (3) Development of a registry which will include HRV parameters measured in a contemporary cohort of patients with STEMI.

### 2.2. Eligibility Criteria

We defined several inclusion criteria which will be applied for each patient:

(1) Age ≥ 18 years;
(2) Patients in sinus rhythm;
(3) STEMI diagnosis referred for primary PCI within 12 h from symptoms onset;
(4) Patients who agree to participate in the study and sign the informed consent.

In addition, we pre-specified key exclusion criteria, which also encompassed factors interfering with RR intervals and HRV parameters:

(1) Patients who are unable to sign the informed consent;
(2) Atrioventricular block of any degree or sinus node dysfunction;
(3) Atrial fibrillation;
(4) Paced ventricular rhythm;
(5) Frequent premature supraventricular or ventricular contractions;
(6) Treatment with positive inotropic or chronotropic drugs;
(7) History of myocardial infarction or myocardial revascularization (PCI or coronary artery bypass graft surgery—CABG);
(8) Patients who refused to participate in the study.

STEMI will be defined according to the fourth universal definition of myocardial infarction [17].

### 2.3. HRV Parameters Measured

We will measure HRV parameters recommended by the ESC and the North American Society of Pacing and Electrophysiology guidelines [7]:

(1) Time domain parameters: standard deviation of all NN intervals (SDNN), HRV triangular index, the standard deviation of the average NN interval over short time divisions (SDANN), and the square root of the mean squared differences of consecutive NN intervals (RMSSD);
(2) Frequency domain parameters: low-frequency power (LF), high-frequency power (HF), and LF/HF ratio.

Additionally, some nonlinear HRV parameters will be evaluated, such as SD1, SD2, SD2/SD1, approximate entropy, and sample entropy.

HRV will be measured in addition to medical and interventional therapy for myocardial infarction according to the latest ESC guidelines focused on STEMI [2]. HRV will be assessed using a medically approved wrist-wearable device (E4 wristband from Empatica, CE certified) for 5 min segments during myocardial revascularization by primary PCI (before and after revascularization). The E4 device will be placed on the non-dominant hand, with the case tightener on the top of the wrist. The validity of HRV measurement using an Empatica E4 wristband was previously confirmed in a clinical study [13]. Raw data regarding heart rate and HRV (interbeat interval data) will be extracted from the wristband and further analyzed using Kubios software. Among available E4 wristband sensors, photoplethysmography will be used to derive interbeat interval data from blood volume pulse analysis. The blood volume pulse signal has a sampling rate of 64 Hz (resolution 1/64 s) as specified by the manufacturer. All HRV parameters of interest (including frequency domain and nonlinear parameters) will be automatically calculated using Kubios software [18]. Moreover, eventual artifacts in raw data collection will be corrected using a very low threshold algorithm (0.45 s) in Kubios to avoid significant HRV parameters deformation. Additional HRV measurements will be performed after the index event (at one and six months).

### 2.4. Collected Data

The following data will be collected and analyzed:

- Demographic data (age, sex);
- Time to primary PCI in relation to chest pain onset;
- Comorbidities (arterial hypertension, diabetes mellitus, chronic kidney disease, ischemic heart disease, stroke);
- Cardiovascular risk factors (advanced age, gender, high body mass index, smoking, sedentarism, inflammation);
- Cardiac rhythm; HRV parameters (time domain, frequency domain, and nonlinear measurements),
- Biological data (creatine kinase-MB—CK-MB, lactate dehydrogenase—LDH, aspartate transaminase—AST, cardiac-specific troponin, complete blood count, hemoglobin, hematocrit, glycemia, lipid profile, serum urea, and creatinine, estimated glomerular filtration rate using CKD-EPI equation, serum potassium, and sodium, C-reactive protein, N-terminal pro-b-type natriuretic peptide);
- Left ventricular ejection fraction (LVEF) evaluated at admission, pre-discharge, and during follow-up;
- Thrombolysis in myocardial infarction (TIMI) flow before and after primary PCI;
- Type of stent used for primary PCI;
- GRACE score;
- SYNTAX score II will be documented if three-vessel coronary disease or left main stem disease.

All data, including patients’ names and the aforementioned data, will be stored in an electronic database available only for study investigators. Collected data in relation to specific timeframes were displayed in Table 1.

### 2.5. Outcomes and Follow-Up

The primary composite outcome will include all-cause mortality and major adverse cardiovascular events (MACE). MACE will include cardiac mortality, fatal and non-fatal myocardial infarction, unplanned target vessel revascularization (TVR), and stroke (ischemic or hemorrhagic).

In addition, several secondary outcomes will be analyzed: (a) all-cause and cardiac mortality; (b) fatal and non-fatal myocardial infarction; (c) unplanned target vessel revascularization; (d) target lesion revascularization (TLR); (e) stroke; (f) hospitalizations for heart failure; (g) ventricular arrhythmias; (h) LVEF and left ventricular diastolic function. Moreover, the primary composite outcome will be evaluated in a high-risk subgroup of patients, such as elderly patients or those with diabetes mellitus/chronic kidney disease.

TVR will be defined as new stenosis requiring PCI or CABG in another part of the coronary artery treated at the index event. TLR will include unplanned PCI or CABG for the same lesion revascularized during primary PCI. LVEF will be measured using two-dimensional transthoracic echocardiography (Simpson methods). At least E/A or E/e prime ratios will be calculated for left ventricular diastolic function. Ventricular arrhythmias will be defined as ventricular fibrillation or sustained ventricular tachycardia documented at electrocardiographic monitoring. The primary composite outcome and secondary outcomes will be evaluated during the hospital stay, at 1 month and 1 year following admission for STEMI. All data regarding the outcomes will be collected from secure medical records.

### 2.6. Participation Timeline

The expected duration of the study is three years following ethical approval by the local ethical review committee. As a first step, eligibility criteria will be analyzed at patients’ admission to the catheterization laboratory. Patients will be enrolled if all inclusion criteria will be fulfilled. During primary PCI procedure, HRV will be continuously monitored using a wearable device, which will be further analyzed offline using Kubios software. Additionally, at the index procedure demographic data, time to primary PCI in relation to chest pain onset; data regarding comorbidities and cardiovascular risk factors; and specified clinical, biological, and echocardiographic parameters will be collected.

During the hospital stay, patients will be re-evaluated echocardiographically and pre-discharge LVEF will be recorded. All outcomes of interest will be reported at this first time point in order to analyze a possible correlation between previously measured HRV parameters and short-term adverse events.

At 1 month and at 12 months after the index event, HRV will be re-assessed. In addition, in these time frames, clinical and echocardiographic data will be collected. Primary and secondary outcomes will be reported and an eventual correlation with HRV measured during primary PCI, as well as after 1 month and 12 months, will be analyzed.

### 2.7. Statistical Analysis

SPSS statistical software version 26.0 will be used (IBM SPSS Statistics, New York, NY, USA). Descriptive statistics will be applied to characterize enrolled patients. Normally distributed data will be represented as mean and standard deviation, while non-normal distributed data will be displayed as median and Inter Quartile Range (IQR). Also, relevant graphics will be used when possible. 

When appropriate, data will be analyzed using parametric (Student’s *t*-test) and non-parametric tests (Mann–Whitney–Wilcoxon). In addition, for statistical hypothesis testing, Student’s *t*-test will be applied in case of continuous variables, while categorical variables will be compared using the chi-square test. Missing data will be handled using a multiple imputation approach.

Odds ratio (OR), corresponding 95% confidence intervals, and p-values will be calculated for each variable related to investigated outcomes. A *p*-value < 0.05 will be considered statistically significant. Univariate logistic regression models will reveal statistically significant variables associated with pre-specified outcomes. Afterward, variables documented as statistically significant in univariate logistic regression models will be included in multivariate analysis. Moreover, the prediction accuracy of time and frequency domain HRV parameters for primary and secondary outcomes will be evaluated using the Receiver Operator Characteristics (ROC) curve. The sensibility, specificity, and c-statistic of individual HRV parameters will be calculated. Additionally, the Kaplan–Meier survival curve will be used to analyze time to event variables.

In addition, the role of HRV parameters for adverse outcomes prediction will be analyzed in three subgroups of high-risk patients, including patients with chronic kidney disease, diabetes mellitus, and elderly participants.

## 3. Results

In order to enhance the reading process, the results will also be displayed as tables and figures. We will provide data regarding HRV parameters values in patients presenting with STEMI. The correlation between HRV and adverse short- and long-term outcomes will be analyzed and presented. The independent value of HRV measurements for predicting primary and secondary outcomes will also be reported.

## 4. Discussion

Our study will prospectively investigate the impact of HRV time domain, frequency domain and nonlinear parameters on adverse outcomes in a contemporary cohort of STEMI patients revascularized by primary PCI. To the best of our knowledge, it will be the first study to document the potential prognostic role of HRV variables in STEMI patients measured with a wrist-wearable medical device.

HRV represents an indirect marker of autonomic nervous system function, reflecting the impact of sympathetic and parasympathetic nervous systems balance on the heart [19]. HRV constitutes not only the result of a complex connection between the central nervous system and the heart, but also an active marker that can be enhanced [20]. In this regard, HRV could be influenced by a unique respiration technique, HRV biofeedback, which could improve outcomes in various chronic conditions, including cardiovascular diseases [21]. As we have previously shown, this bidirectional interaction is a dynamic process, while modulating HRV could have favorable implications on STEMI end points [22].

Consequently, this expression of heart–brain interaction could represent a prognostic marker in patients with cardiovascular diseases [23]. A meta-analysis on 28 cohort studies involving patients with cardiovascular diseases reported that HRV parameters (both time- and frequency domain variables) were significantly associated with all-cause death (HR 2.12, 95% CI, 1.64–2.75) and adverse cardiovascular events (HR 1.46, 95% CI, 1.19–1.77). Interestingly, in a subgroup analysis, HRV values were only correlated with all-cause death in the case of patients with AMI (HR 2.52, 95% CI, 1.75–3.62), but not in those with heart failure (HR 1.47, 95% CI, 0.99–2.17) [24]. However, studies that enrolled AMI patients are old and did not reflect current treatment options and guidelines. As a consequence, the impact of HRV measurement in contemporary patients with STEMI should be reassessed.

In addition to its possible prognostic value, HRV might be used for AMI diagnosis and myocardial ischemia detection. In one study which enrolled patients without known coronary artery disease, a low HRV had an excellent negative predictive value for myocardial ischemia (97%), though sensitivity and specificity were lower (respectively, 71% and 60%) [25]. In another study, the authors reported that low HRV values were significant predictors for obstructive coronary artery disease, even after multivariable adjustment [10]. Moreover, we will try to elucidate the role of HRV assessment for secondary prevention following STEMI by investigating the incidence of MACE, including fatal and non-fatal myocardial infarction and TLR.

HRV was established as a prognostic marker in the case of patients with end-stage kidney disease undergoing hemodialysis. In this regard, a recent meta-analysis showed increased all-cause mortality (HR 1.63, 95% CI, 1.11–2.39) linked, mainly, to lower SDANN and LF/HF ratio values [5]. Similar results are expected to be documented in the case of patients presenting with STEMI.

Instantaneous HRV assessment could be influenced by various intrinsic and environmental factors. Although short-term and ultrashort-term HRV parameters were investigated extensively investigated in clinical studies, these values should not be used interchangeably with long-term 24 h parameters [11].

In the last years, more data became available in favor of HRV measurement with photoplethysmography using various wrist-worn devices [13,26]. These devices could provide an accurate assessment of all HRV parameters, including time domain, frequency domain, and nonlinear parameters. Usually, wearable devices could collect interbeat interval data, further analyzed with software to derive all HRV parameters [13,26]. Hence, we will investigate the utility of wrist-worn-device-derived HRV parameters to identify patients with STEMI at a higher risk of worse outcomes. Additionally, we will provide an eventual tool for the residual risk stratification in AMI patients and LEFV recovery prediction [27]. In addition, the present study will represent a background to support the integration of HRV parameters in remote control algorithms in this subset of patients as a simple and noninvasive measurement.

## 5. Conclusions

HRV measurement represents a feasible and easy-to-measure marker due to technological progress. Our study will enlighten the reliability and usefulness of HRV evaluation as a prognostic marker in contemporary patients with STEMI. The potential validation of HRV as a risk marker for adverse outcomes following STEMI will ensure a background for including HRV parameters in future risk scores and guidelines. In addition, we will highlight the importance of new methods of HRV assessment, such as photoplethysmography using wrist-worn devices.

## Figures and Tables

**Table 1:** Collecting data at specific time points.

Procedures/Timepoint	Study Interventions				
	Enrollment	Baseline	During Hospital Stay	1 Month	12 Months
**Enrollment**	X				
Screen for eligibility	X				
Contact information	X				
Informed consent					
**Interventions**					
Demographic data		X			
Time to PCI		X			
Comorbidities		X			
Cardiovascular risk factors		X			
Blood collection		X			
Echocardiography		X	X	X	X
Angiographic data		X			
ECG		X	X	X	X
HRV measurement		X		X	X
**Assessments**					
Clinical data		X	X	X	X
Primary composite outcome			X	X	X
Secondary outcomes			X	X	X

ECG = electrocardiography; HRV = heart rate variability; PCI = percutaneous coronary intervention.

## Supplementary Materials

The following supporting information can be downloaded at: https://www.mdpi.com/article/10.3390/s22093571/s1, Table S1: STROBE statement.
